# Species-specific dehydration tolerance and its measurement comparison in drosophilids of Western Himalayas

**DOI:** 10.3389/fphys.2022.880684

**Published:** 2022-08-17

**Authors:** Divya Singh, Seema Ramniwas

**Affiliations:** University Center for Research and Development, Chandigarh University, Gharuan, India

**Keywords:** *Drosophila* species, Western Himalayas, dehydration tolerance, desiccation resistance, comparision

## Introduction

Insects’ ability to tolerate dehydration stress is understudied, yet important. The challenges of desiccating conditions confront insects in arid habitats, which can be overcome by one of four novel adaptive mechanisms: 1) reducing the rate of water loss (Gibbs et al., 1997), 2) increasing bulk water, 3) changing the phospholipid composition of epicuticular lipids (Rourke, 2000), or 4) tolerating greater amounts of water loss, that is, dehydration tolerance, (Hoffmann and Parsons, 1989; Gibbs et al., 1997; Gibbs and Matzkin, 2001). While there are several studies on dehydration tolerance and its role in desiccation resistance (Strachan et al., 2015; Weldon et al., 2016; Thorat and Nath, 2018; Ajayi et al., 2020), its significance remains controversial and there are no direct supporting evidence. It has not been extensively studied that dehydration tolerance could influence the evolution of desiccation resistance, but this aspect may play a role in diverse insect taxa.

Water deficit environments can cause organisms to use different survival mechanisms. These include avoiding water loss or tolerating it. The tolerance to dehydration in dry environments consists of being able to survive internal water deficits (Scott, 2000), while dehydration avoidance is just the opposite (Pallarés et al., 2016). It is argued that avoiding dehydration should be a priority strategy, but the former lacks adequate data. Dehydration tolerance has been examined by a limited number of studies (Supplementary Data Sheet S1, Sheet 2-4), and most of those studies used selected *D. melanogaster*. *Drosophila* species adapted to desert conditions are more resistant to dehydration than their mesic counterparts (Gibbs and Matzkin, 2001); however, laboratory-selected strains showed no differences in resistance to desiccation (Gibbs et al., 1997). Comparatively, Hoffmann and Parsons (1993) reported that D (desiccation induced) flies were more dehydrated than their control group. Laboratory selection experiments generally assumed that desiccation resistance is not enhanced by dehydration tolerance (Gibbs et al., 1997). The term “dehydration tolerance” has been a topic of controversy for a long time, and the difference in terminology used by different scientists might explain this. The discrepancy in calculating dehydration tolerance might have resulted as a consequence of different formulas used by different physiologists. Table 1 shows the different formulas that were used for calculation of dehydration tolerance.

**TABLE 1:** Summary of calculations used from different physiologists for estimating dehydration tolerance.

Assumption for Standardising the formula				
•Initial body weight (a) = 1.475 mg × 10^3^			**Total water content (%) = {(a-c)/a} × 100 = 69.22 %**	
•Weight after drying (b) = 0.454 mg × 10^3^				
•Weight at death (c) = 1.033 mg × 10^3^				

	Formulae used	Outcome	Calculated value	Reference
1.	b−c	Water remaining after death	0.579 or 57.9 %	Hoffmann and Parsons (1993)
2.	(a−c)/(a−b)	Water lost at death out of total water content	0.433 or 43.3 %	Gibbs et al. (1997)
3.	(b−c)/a	Water content at death	0.393 or 39.3 %	Brisson et al. (2005)

There is limited evidence for simultaneous analysis of stress-related traits across sympatric populations of different species of *Drosophila* on a continent, with contrasting distribution patterns. This report compares wild species from Western Himalayas to explore the relative importance of dehydration tolerance as a strategy to desiccation resistance. It is possible to explain differences in desiccation resistance between species based on dehydration tolerance, while water loss rates, cuticular melanization, and epicuticular lipid levels do not explain these differences. Desiccation resistance and their adaptation to different habitats are thus influenced by species-specific differences in dehydration tolerance.

## Material and methods

### Collections

Table 2 shows different *Drosophila* species collected in autumn (October 2018) from five altitudinal localities of Western Himalayas using net sweeping and bait trap methods. These *Drosophila* species were categories as cold adapted, generalist, and warm adapted according to their thermal range (12–32°C) of development and distribution pattern (shown in Supplementary Data Sheet S1, Sheet 1). Cultures were maintained at 21°C on cornmeal yeast agar medium based on T_ave_ data obtained from Indian Institute of Tropical Meteorology (IITM; www.tropmet.res.in) of the sites of origin of populations under the photoperiod of 12 h dark and 12 h light. As the trait values did not vary between 6 and 21 days (Gibbs and Matzkin, 2001; Parkash et al., 2008), all assays were performed on 7 days old female flies of wild caught—G_1_ only.

**TABLE 2:** Number of individuals of different species of *Drosophila* collected in autumn (October, 2018).

Species/Altitude (m)	Chandigarh (347)	Parwanoo (470)	Kalka (600)	Solan (1440)	Shimla (2202)
***Cold adapted species***					
*Drosophila nepalensis*	30	33	33	67	320
*Drosophila buskii*	-	02	198	275	303
*Drosophila simulans*	29	35	176	234	285
*Drosophila hydei*	18	19	35	400	375
*Drosophila punjabiensis*	15	20	23	45	62
***Generalist species***					
*Drosophila melanogaster*	270	273	225	96	2
*Drosophila kikkawai*	72	89	36	53	47
***Warm adapted species***					
*Drosophila biarmipes*	130	136	6	8	-
*Drosophila nasuta*	250	83	19	-	-
*Drosophila parabipectinata*	25	29	-	-	-
*Drosophila bipectinata*	16	15	03	-	-
*Zaprionus Indianus*	263	202	135	50	01
*Drosophila ananassae*	335	401	120	53	3

### Trait measurements

Olympus magnifying instrument with stereo-zoom specification SZ-61 was used to score body melanization of females. Only females were measured because of their variable abdominal sections for body melanization as contrasted with males where the last two portions are dark. For every one of the six abdomen fragments (second-seventh) which vary in size (i.e., 0.86, 0.94, 1.0, 0.88, 0.67, and 0.38 for second to seventh fragments, individually) from dorsal and sidelong perspectives, 0 (no melanization) to 10 (complete melanization) qualities were assessed. % melanization = $\frac{\Sigma \;\mathrm{Melanization scores of abdominal sections per}\;\mathrm{fly}}{\mathrm{\Sigma relative size of every abdominal fragment}}\;\times 100$ (Parkash et al., 2008).

### Resistance to desiccation stress

Resistance to desiccation stress was estimated as the time to deadly lack of hydration impact under dry air. For this isolation of individual females of various locations was carried out in a vial containing silica gel (2 g). These vials were set in a desiccator (having 6–8% RH). Total stationary flies were tallied after each 1 h interim, and the timeframe to deadly drying up impact (LT_100_) was recorded.

### Water balance measures

Microbalance from Sartorius was used to weigh singular grown-up flies of each location for deciding wet weight (a) and dry weight (b) and absolute water (a-b). Every fly from that point in the Eppendorf tube was dried overnight at 60°C & reweighed after drying. Total body water content = mass before drying−mass after drying  , and when this total body water is lost because of drying up (until death) then dehydration tolerance was estimated by the equation $\frac{a-\mathrm{weight}\;\mathrm{at}\;\mathrm{death}\left(c\right)}{a-b}\times 100$ (Gibbs et al., 1997). Previously tested formulas by different physiologists (Hoffmann and Parsons, 1993; Gibbs et al., 1997; Brisson et al., 2005; Parkash et al., 2008) were also compared to eliminate the discrepancy resulting from different definitions and formulas used for dehydration tolerance.

Cuticular water loss due to short term desiccation (8 h) was also carried out. Flies were weighed both before and after desiccation, and the cuticular water loss was calculated (in mg hr^−1^) as $\frac{a–\mathrm{weight}\;\mathrm{after}\;8\;\mathrm{hr}\;\mathrm{of}\;\mathrm{desiccation}\;\mathrm{stress}}{a}\times 8$ .

### Statistical analyses

For all the traits (body melanization, desiccation resistance, cuticular water loss, and dehydration tolerance) population means (*n* = 5 locations × 10 individuals × 10 replicates) of one cold adapted (*Drosophila nepalensis*) one generalist (*Drosophila melanogaster*) and one warm adapted species (*Drosophila ananassae*) along with SE were used for illustrations and tabular data. Trait variability was analyzed using ANOVA. Statistical calculations and illustrations were made with the help of Statistica™ 7.0.

### Data description

**FIGURE 1:**
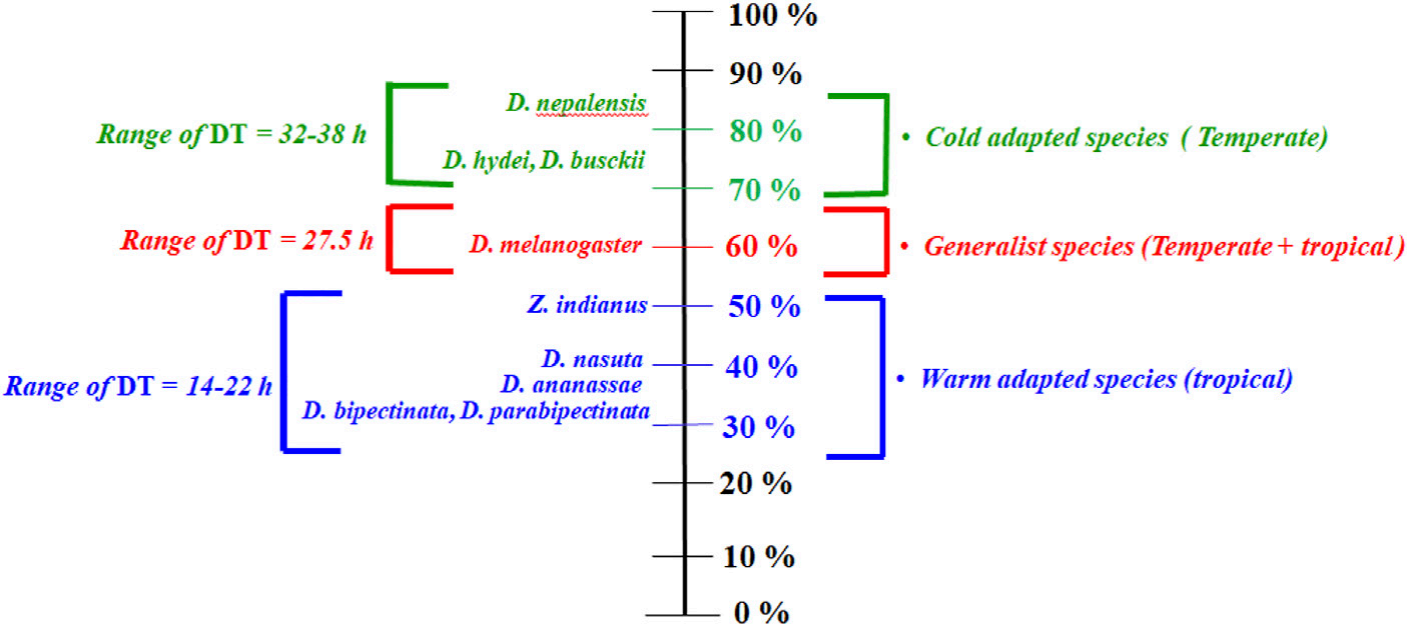
Scale for categorizing *Drosophila* species according to their dehydration tolerance. The 0% shows a lack of desiccation tolerance, while the 100% shows great desiccation tolerance. Range of DT is resistance to desiccation (in hours) of these species.

A common garden experiment was used to illustrate population differences since wild populations are adapted to different local climates. It minimized environmental effects and demonstrated genetic differences between populations. Interesting results are found for desiccation resistance (hours); cold-adapted species have a resistance to desiccation twice as high as generalist and warm-adapted species. (Figure 1). For desiccation survival hours and dehydration tolerance, there are contrasting differences between species, that is, *p* < 0.001, Figure 1. There were not significant differences in rate of water loss of these species that could account for variable desiccation hours (Supplementary Data Sheet S1); however, cold adapted species have slightly lower rate of water loss.

Water balance characteristics for *D. n*, *D. m*, and *D. a* are represented in Table 3. Total water content (∼68%) did not vary between populations as well as species.

**TABLE 3:** Data (mean ± SE) on basic measures of water balance related traits (a) and dehydration tolerance (b) in Cold adapted (*D. n)*, generalist (*D. m)* and warm adapted (*D.a)* species.

	(a) Basic measures of water (mg fly^−1^)			(b) Different measures of dehydration tolerance (mg fly^−1^)		
Species/Traits	Wet mass	Dry mass	Total water content	Water remain at death	Water lost under desiccation	Dehydration tolerance (%)
Cold adapted (*D. n*)	1.605^b^ ± 0.73	0.481^b^ ± 0.41	1.124^b^ ± 0.07	0.208 ± 1.78	0.925 ± 2.01	82.3^b^
Generalist (*D. m*)	1.550^a^ ± 1.01	0.465^a^ ± 0.08	1.085^a^ ±0.12	0.434 ± 0.29	0.651 ± 0.51	60.0^a^
Warm adapted (*D. a*)	1.319^c^ ± 0.35	0.396^c^ ± 0.01	0.923^c^ ± 0.05	0.575 ± 0.51	0.348 ± 0.29	37.7^c^
*F*_4,496_	5326.48***	7994.22***	842.56***	4249.56***	3568.91***	3446.78***

***p < 0.001 indicate significant differences based on ANOVA and post- hoc comparisons are shown as letters in superscripts indicating pair wise comparison. The values with different superscript letters in a column are significantly different (*p* < 0.005).

It has been established that desiccation resistance and dehydration tolerance are correlated positively (*D. n*: r = 0.83 ± 0.05; *p* < 0.001; *D. m*: r = 0.79 ± 0.07; *p* < 0.001; *D. a*: r = 0.80 ± 0.03; *p* < 0.001). With respect to body melanization and cuticular lipids, correlation values are non-significant, and therefore, desiccation resistance does not appear to be related to these traits. Contrary to this, correlations between desiccation resistance and dehydration tolerance are very significant (*p* < 0.001). As a result, different levels of dehydration tolerance predict different desiccation survival times. A higher survivability at 0% RH is apparent from cold-adapted species’ greater dehydration tolerance.

## Discussion

In order to cope with desiccating conditions, insects have developed many morphological and physiological adaptations. Insects can survive water loss if they have dehydration tolerance.

Previous comparative studies of *Drosophila* species have generally considered species that are differing in their cuticular melanization, epicuticular lipids, and/or rate of water loss. Compared with all of these factors, role of dehydration tolerance in desiccation resistance comes out to be small or negligible. In this study, our focus is on species comparison that are not differing in these factors to find out the possible physiological modification that help them to endure different habitats and to work out weather dehydration tolerance is really helpful. The present study has shown that the tolerance level toward dehydration can explain changes in desiccation resistance levels (Figure 1).

Based on basic water balance characteristics, most of the insects have about 66–68% water (Hadley, 1994) and similar results were obtained in this study (water content ∼66.89–72.22%). The comparison of previously used formulas was performed by using same water content values (Table 3) to come out at a more definite conclusion hence to solve the problem regarding dehydration tolerance. Dehydration tolerance was described as the body water loss prior to death (Brisson et al., 2005; Telonis-Scott et al., 2006; Archer et al., 2007); however, calculations used by Hoffmann and Parsons (1993) depicted the remaining water content at the time of death. The formula given by Gibbs et al. (1997) is more accurate as it depicts actual dehydration tolerance, that is, amount of water lost at death out of total amount of body water content. Cold adapted species lose a greater fraction of their total body water content before they die (∼57%), whereas generalist species died after losing 42% of their water content, and warm adapted species die by losing only 26% of water content.

The magnitude of desiccation resistance varies across different *Drosophila* species (Gibbs and Matzkin, 2001). Varying resistance levels among species are often linked with their distribution patterns, but the target of natural selection is not clear. Significant differences occur for dehydration tolerance of various species. Such data have supported the role of dehydration tolerance in conferring desiccation resistance in these species. Thus, species that tend to occupy relatively drier habitats are able to withstand greater dehydration tolerance.

The present study concludes that species-specific changes in dehydration tolerance are associated with changes in desiccation resistance. In warm-adapted species, a lower percentage of dehydration tolerance is associated with a lower level of desiccation resistance. The abundance of generalist species in sub-tropical habitats is a reflection of the humid conditions in such habitats, and its distribution decreases as latitude increases. Conversely, cold-adapted species with high levels of dehydration tolerance may sustain excessive water losses under drier climatic conditions and, as a result, occur along the Indian subcontinent’s high altitudes. *Drosophila* species show varying levels of dehydration tolerance, which may affect their resistance to desiccation. Until now, dehydration tolerance, despite its importance to physiological survival under dry conditions, has not been sufficiently studied. In light of current findings, this view has to be revised, and the ecology and ecology implications need to be reconsidered so that under climate change understanding the role of dehydration tolerance for survival under desiccating condition could be understood because it might increase successful invasion of various pest species in arid habitat.

## Data Availability

The original contributions presented in the study are included in the article/Supplementary Materials; further inquiries can be directed to the corresponding author.

## References

[B1] Ajayi O. S. Appel A. G. Chen L. Fadamiro H. Y. (2020). Comparative cutaneous water loss and desiccation tolerance of four Solenopsis spp. (hymenoptera: Formicidae) in the southeastern United States. Insects 11, E418. 10.3390/insects11070418 PubMed Abstract | 10.3390/insects11070418 | Google Scholar 32635677 PMC7412113

[B2] Archer M. A. Bradley T. J. Mueller L. D. Rose M. R. (2007). Using experimental evolution to study the physiological mechanisms of desiccation resistance in *Drosophila melanogaster* . Physiol. Biochem. Zool. 80, 386–398. 10.1086/518354 PubMed Abstract | 10.1086/518354 | Google Scholar 17508334

[B3] Brisson J. A. De Toni D. C. Duncan I. Templeton A. R. (2005). Abdominal pigmentation variation in Drosophila polymorpha: Geographic variation in the trait, and underlying phylogeography. Evolution 59, 1046–1059. 10.1111/j.0014-3820.2005.tb01043.x PubMed Abstract | 10.1111/j.0014-3820.2005.tb01043.x | Google Scholar 16136804

[B4] Gibbs A. G. Chippindale A. K. Rose M. R. (1997). Physiological mechanisms of evolved desiccation resistance in *Drosophila melanogaster* . J. Exp. Biol. 200, 1821–1832. 10.1242/jeb.200.12.1821 PubMed Abstract | 10.1242/jeb.200.12.1821 | Google Scholar 9225453

[B5] Gibbs A. G. Matzkin L. M. (2001). Evolution of water balance in the genus Drosophila. J. Exp. Biol. 204, 2331–2338. 10.1242/jeb.204.13.2331 PubMed Abstract | 10.1242/jeb.204.13.2331 | Google Scholar 11507115

[B6] Hadley N. F. (1994). Water relations of terrestrial arthropods. Available at: https://www.elsevier.com/books/water-relations-of-terrestrial-arthropods/hadley/978-0-08-091852-5 . Google Scholar

[B7] Hoffmann A. A. Parsons P. A. (1993). Direct and correlated responses to selection for desiccation resistance: A comparison of *Drosophila melanogaster* and *D. simulans* . J. Evol. Biol. 6, 643–657. 10.1046/j.1420-9101.1993.6050643.x 10.1046/j.1420-9101.1993.6050643.x | Google Scholar

[B8] Hoffmann A. A. Parsons P. A. (1989). Selection for increased desiccation resistance in *Drosophila melanogaster*: Additive genetic control and correlated responses for other stresses. Genetics 122, 837–845. 10.1093/genetics/122.4.837 PubMed Abstract | 10.1093/genetics/122.4.837 | Google Scholar 2503423 PMC1203758

[B9] Pallarés S. Velasco J. Millán A. Bilton D. T. Arribas P. (2016). Aquatic insects dealing with dehydration: Do desiccation resistance traits differ in species with contrasting habitat preferences? PeerJ 4, e2382. 10.7717/PEERJ.2382 PubMed Abstract | 10.7717/PEERJ.2382 | Google Scholar 27635346 PMC5012287

[B10] Parkash R. Ramniwas S. Rajpurohit S. Sharma V. (2008). Variations in body melanization impact desiccation resistance in *Drosophila immigrans* from Western Himalayas. J. Zoology 276, 219–227. 10.1111/j.1469-7998.2008.00478.x 10.1111/j.1469-7998.2008.00478.x | Google Scholar

[B11] Rourke B. C. (2000). Geographic and altitudinal variation in water balance and metabolic rate in a California grasshopper, *Melanoplus sanguinipes* . J. Exp. Biol. 203, 2699–2712. 10.1242/jeb.203.17.2699 PubMed Abstract | 10.1242/jeb.203.17.2699 | Google Scholar 10934009

[B12] Scott P. (2000). Resurrection plants and the secrets of eternal leaf. Ann. Bot. 85, 159–166. 10.1006/anbo.1999.1006 10.1006/anbo.1999.1006 | Google Scholar

[B13] Strachan S. R. Chester E. T. Robson B. J. (2015). Freshwater invertebrate life history strategies for surviving desiccation. Springer Sci. Rev. 3, 57–75. 10.1007/s40362-015-0031-9 10.1007/s40362-015-0031-9 | Google Scholar

[B14] Telonis-Scott M. Guthridge K. M. Hoffmann A. A. (2006). A new set of laboratory-selected *Drosophila melanogaster* lines for the analysis of desiccation resistance: Response to selection, physiology and correlated responses. J. Exp. Biol. 209, 1837–1847. 10.1242/jeb.02201 PubMed Abstract | 10.1242/jeb.02201 | Google Scholar 16651550

[B15] Thorat L. Nath B. B. (2018). Insects with survival kits for desiccation tolerance under extreme water deficits. Front. Physiol. 9, 1843. 10.3389/fphys.2018.01843 PubMed Abstract | 10.3389/fphys.2018.01843 | Google Scholar 30622480 PMC6308239

[B16] Weldon C. W. Boardman L. Marlin D. Terblanche J. S. (2016). Physiological mechanisms of dehydration tolerance contribute to the invasion potential of Ceratitis capitata (Wiedemann) (Diptera: Tephritidae) relative to its less widely distributed congeners. Front. Zool. 13, 15. 10.1186/s12983-016-0147-z PubMed Abstract | 10.1186/s12983-016-0147-z | Google Scholar 27034703 PMC4815119

